# Transcriptome analyses suggest a novel hypothesis for whitefly adaptation to tobacco

**DOI:** 10.1038/s41598-017-12387-3

**Published:** 2017-09-21

**Authors:** Wen-Qiang Xia, Xin-Ru Wang, Yan Liang, Shu-Sheng Liu, Xiao-Wei Wang

**Affiliations:** Ministry of Agriculture Key Laboratory of Agricultural Entomology, Institute of Insect Sciences, Zhejiang University, Hangzhou, 310058 China

## Abstract

The adaptation of herbivorous insects to various host plants facilitates the spread and outbreak of many important invasive pests, however, the molecular mechanisms that underneath this process are poorly understood. In the past three decades, two species of the whitefly *Bemisia tabaci* complex, Middle East-Asia Minor 1 and Mediterranean, have invaded many countries. Their rapid and widespread invasions are partially due to their ability to infest a wide range of host plants. In this study, we determined the transcriptome and phenotypic changes of one Mediterranean whitefly population during its adaptation to tobacco, an unsuitable host plant. After several generations on tobacco, whiteflies showed increased survival and fecundity. High-throughput RNA sequencing showed that genes involved in muscle contraction and carbohydrate metabolism were significantly up-regulated after adaptation. Whiteflies reared on tobacco were further found to have increased body volume and muscle content and be trapped by tobacco trichomes in a lower frequency. On the other hand, gene expression in endosymbionts of whitefly did not change significantly after adaptation, which is consistent with the lack of cis-regulatory element on endosymbiont genomes. Over all, our data suggested that higher body volume and strengthened muscle might help whiteflies overcome physical barriers and survive on tobacco.

## Introduction

Rather than acting as passive victims, plants have developed a variety of mechanisms to promote resistance against herbivorous insects. Physical barriers, such as leaf surface waxes, thorns or trichomes and tissue thickness, perform as the primary obstacles to deter the insects from feeding. And plants’ secondary metabolites can act as toxins affecting growth, development, and digestion of insects^1–3^. Nevertheless, insects can always develop behavioral and physiological mechanisms to counteract the effects of plant defenses, such as avoidance, excretion, and degradation of the toxins^4,5^. Studying the adaptation of insect to plant’s defenses can help us to better understand the plasticity of insect and develop pest control strategies.

The whitefly *Bemisia tabaci* (Gennadius) (Hemiptera: Aleyrodidae) is a genetically diverse species complex including many morphologically indistinguishable cryptic species^6^. In this species complex, the Mediterranean (MED, previously referred as the ‘Q biotype’) and the Middle East-Asia Minor 1 (MEAM1, previously referred as the ‘B biotype’) whiteflies are well known due to their worldwide invasions and displacements of indigenous whiteflies^6^. MED whitefly was first detected in China in 2003, and then expanded its range to most regions of China^7^. Field survey revealed a significant change in the abundance of MEAM1 and MED from 2003 to 2009 in China, suggesting that MED had displaced the earlier invader MEAM1 in many regions^8^.

The invasion by alien species and competition between species are mediated by many biotic and abiotic factors. Previously, asymmetric mating interactions between MEAM1 and indigenous whiteflies have been described to help MEAM1 invasion^9^. The beneficial interactions between begomoviruses and whiteflies may help the invasion of their insect vectors^10^. And the lower susceptibility of invasive whiteflies to commonly used insecticides may drive the competition in favor of them in the field^11,12^. In addition, the range of host plants and host adaptability of different whitefly cryptic species may also play important roles during whitefly invasion and displacement^13^. Comparison between MEAM1 and an indigenous whitefly, Asia II 3, showed that MEAM1 performed much better than Asia II 3 on cotton, tobacco, cabbage, squash, and kidney bean. Asia II 3 whitefly even cannot develop successfully from egg to adult on tobacco, whereas MEAM1 can develop on it with low suitability^14^. On the other hand, MED and ZHJ2, another indigenous whitefly, perform better than MEAM1 on pepper^15^. Different capacity to survive on various host plants is important in mediating the process of whitefly invasion^14,15^. Thus, it is of interest to examine how invasive whitefly adapt to an unsuitable host plant.

To date, several studies investigating the short-term responses of whitefly to unsuitable host plants have been reported. On the unsuitable tobacco plant, the activity of oxidative phosphorylation, citrate cycle, glycolysis and detoxification were repressed in the indigenous Asia II 3 whitefly; however, these pathways were not repressed in the invasive MEAM1^16^. Wang *et al*.^17^ further showed that invasive whiteflies could perform better than indigenous whiteflies on tobacco due to their high activity of detoxification. However, these studies only reported the short-term response of whitefly to unsuitable host plants. Whether and how whiteflies were able to improve their performance on tobacco after several generations’ adaptation remain unclear. Long-term adaptation to unsuitable host plant may cause not only physiological acclimatization but also genetic adaptation. We speculated that there might be some changes that cannot be observed in short-term experiment and make invasive whiteflies adapt to tobacco plant.

In this study, we separately raised MED whiteflies on cotton (a very suitable host) and tobacco (an unsuitable host) plants for more than 10 generations. We found that MED whiteflies could adapt to tobacco plant in a few generations. In order to explore the molecular mechanism underlying this process, gene expression profiles of the two MED populations were compared through RNA sequencing. We found that long-term stress from tobacco plant could reshape the gene expression profiles of MED whitefly and cause changes that cannot be observed in the short-term experiment.

## Results

### Adaptation of whitefly to tobacco

**Figure 1:**
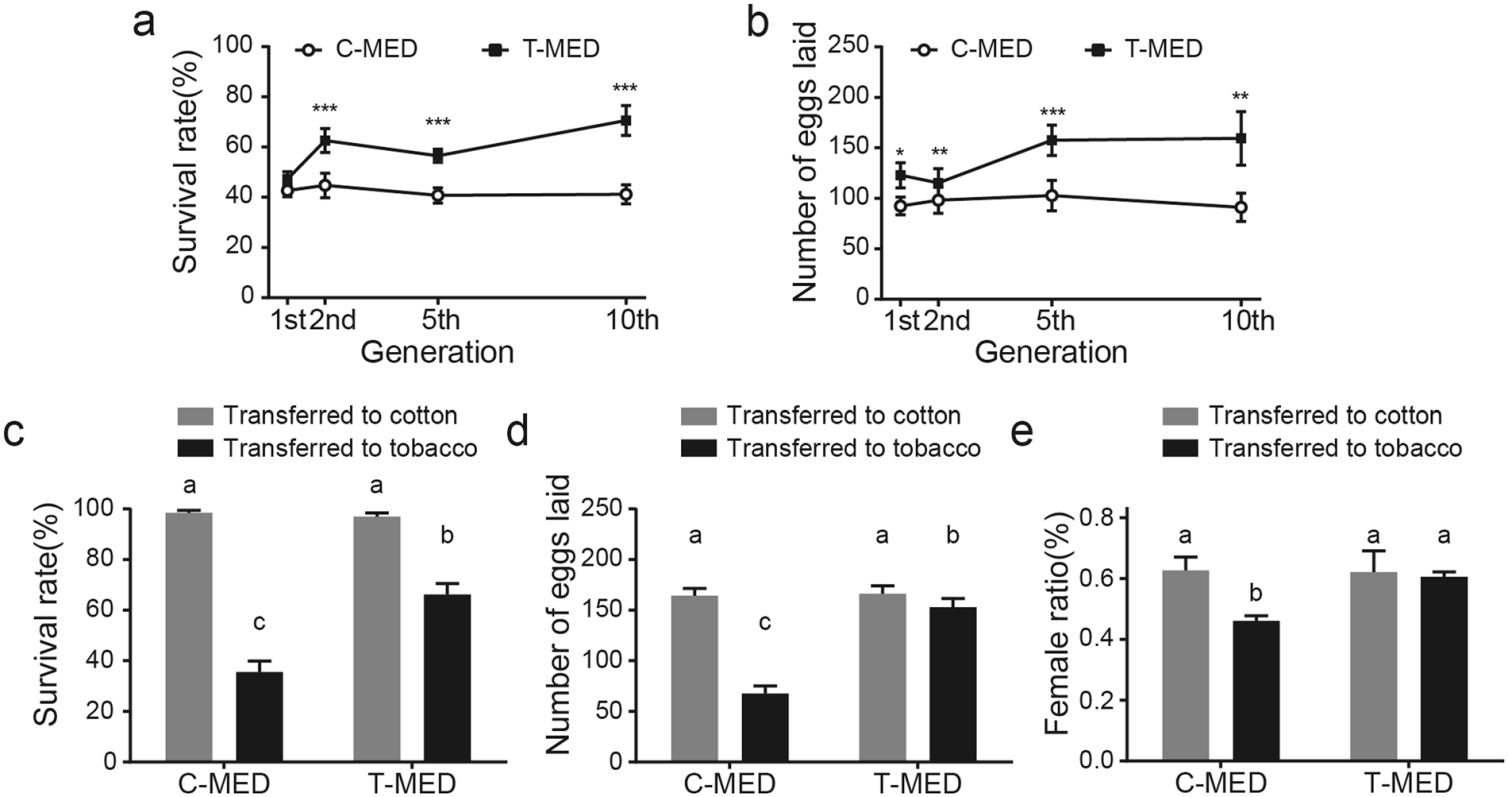
The performance of two whitefly populations on tobacco. After rearing on cotton or tobacco for different generations, whiteflies were transferred to new tobacco plants. Then, the survival rate (**a**) and fecundity (**b**) of the two populations on tobacco were examined. After adaptation for more than 10 generations, survival rate (**c**), fecundity (**d**), and female ratio (**e**) of the two populations were tested on cotton and tobacco. Data shown are mean ± SE. n = 20. Statistically significant difference: *P < 0.05, **P < 0.01, ***P < 0.001. Different letters indicate significant differences between treatments at P < 0.05.

A MED whitefly culture in the laboratory was transferred to tobacco and cotton plants and raised separately. After the whitefly populations had been separately maintained on tobacco (T-MED) or cotton (C-MED) for 1, 2, 5 and 10 generations, the performances of both populations were evaluated on new tobacco plants. Since the second generation, the T-MED showed higher fecundity and survival rate than the C-MED on tobacco plants (Fig. 1a,b). The slow increase of T-MED’s survival rate between the fifth and tenth generation indicated that T-MED might have adapted to tobacco plants. In contrast, there were no significant changes in the performances of C-MED among different generations.

Next, we collected T-MED and C-MED populations that had been separately reared for more than 10 generations and compared their performances on both tobacco and cotton plants respectively. Cotton is a suitable host plant for whitefly, and both populations showed high and similar survival rate, fecundity, and female ratio on cotton (Fig. 1c–e). Tobacco plant caused severe decrease in survival rate, fecundity, and female ratio of C-MED; however, it had less impact on T-MED (Fig. 1c–e). These results suggested that MED whitefly could adapt to tobacco plant in several generations and the adaptation to tobacco would not influence their performances on cotton plant.

### RNA sequencing and differentially expressed genes

To reveal how MED whitefly adapt to tobacco plant, C-MED and T-MED that had been maintained for 10 generations were collected and sequenced, respectively. A summary of sequencing and mapping is shown in Table 1. A total of 65.5% reads from T-MED and 59.5% reads from C-MED were mapped to whitefly genome (Table 1). And about 1.5% reads of T-MED and 2.4% reads of C-MED were aligned to the genome of *Portiera* (obligate endosymbiont). 0.3% reads of T-MED and 0.3% reads of C-MED were aligned to the genome of *Hamiltonella* (facultative endosymbiont) (Table 1).

**Table 1:** Summary for transcriptome sequencing of MED whiteflies reared on cotton and tobacco.

Feature	T-MED^1^	C-MED^2^
Total number of raw reads	223,724,184	217,016,044
Total number of clean reads	162,646,808	185,981,754
Average read length	100	100
Q30^3^ (%)	86.12	88.56
Reads aligned once to *Portiera*	2,471,096 (1.5%)	4,496,078 (2.4%)
Reads aligned more than once to *Portiera*	4,492 (0.0%)	8,500 (0.0%)
Reads aligned once to *Hamiltonella*	442,192 (0.3%)	550,732 (0.3%)
Reads aligned more than once to *Hamiltonella*	4,708 (0.0%)	5,780 (0.0%)
Reads aligned once to whitefly	106,481,554 (65.5%)	110,718,798 (59.5%)
Reads aligned more than once to whitefly	7,865,766 (4.8%)	8,868,234 (4.8%)

^1^The MED whitefly reared on tobacco for 10 generations.

^2^The MED whitefly reared on cotton for 10 generations.

^3^Bases of which PHRED score is greater than 30.

A total of 151 genes were differentially expressed between the two whitefly populations, of which 59 genes were up-regulated and 92 genes were down-regulated in T-MED (Supplementary Table S1). The fold changes (log_2_ ratios) of differentially expressed genes ranged from −2.914 to 2.845. The top up-regulated genes included purine nucleoside phosphorylase, cuticle protein 6, cuticle protein 1 and flightin. The top down-regulated genes included thaumatin-like protein 1a, squalene-hopene cyclase, 5′-nucleotidase and acid phosphatase-1. To validate the reliability of gene expression difference, T-MED and C-MED populations were collected again and their gene expression level were confirmed with RT-qPCR. 10 up-regulated, 10 down-regulated and 10 non-differentially expressed genes were selected randomly and three biological replications were done for each population. The RT-qPCR results were consistent with transcriptome analyses, providing further evidence for the reliability of sequencing data (Supplementary Fig. S1).

### GO enrichment analysis

**Figure 2:**
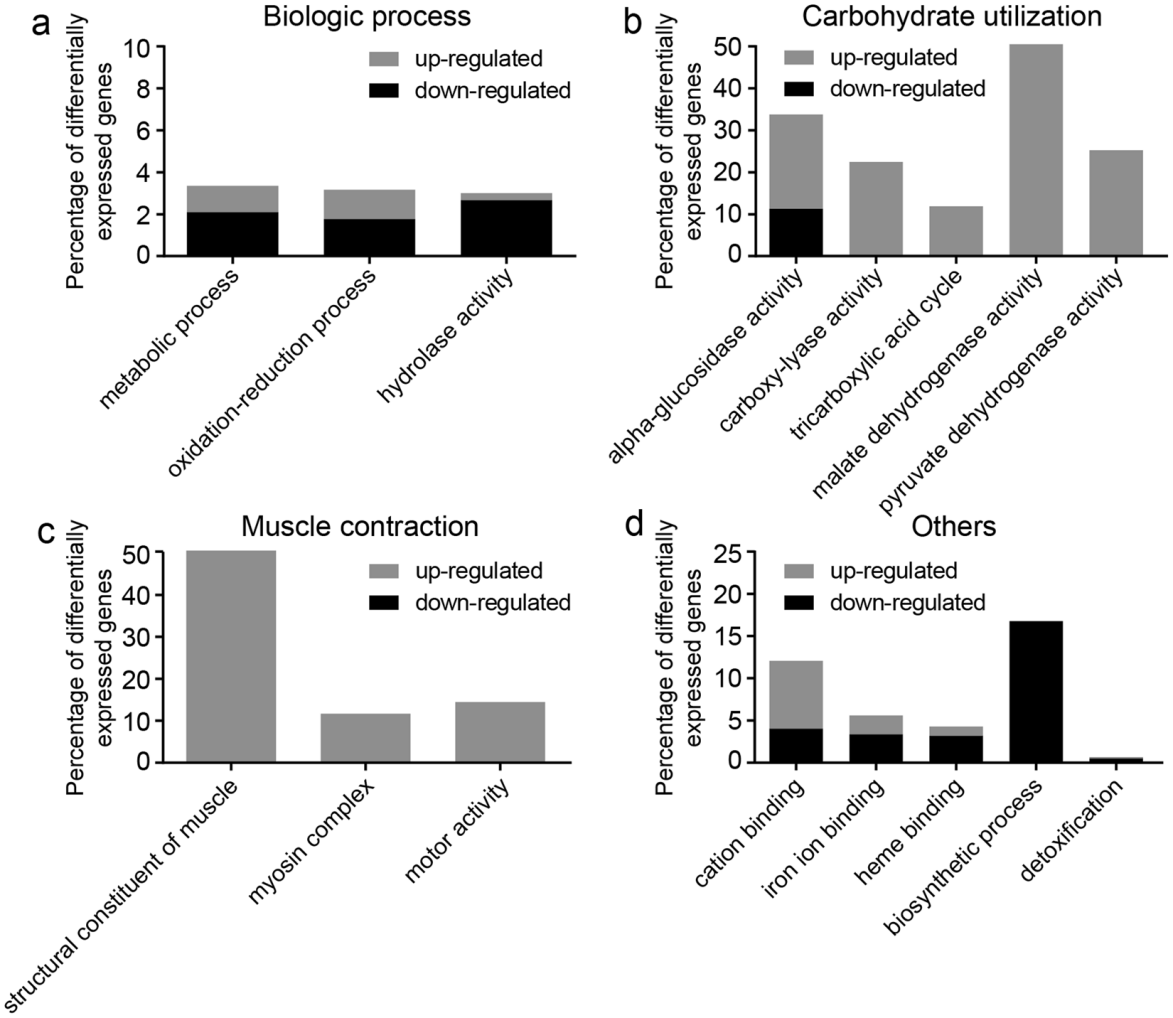
Gene expression profile of whitefly. Regulated genes involved in the biologic process (**a**), carbohydrate utilization (**b**), muscle contraction (**c**), and others (**d**). A large portion of genes involved in muscle contraction and metabolism of carbohydrate were up-regulated after rearing on tobacco for 10 generations.

GO enrichment analysis revealed that the expression of genes in the metabolic process, troponin complex, alpha-glucosidase activity and myosin complex underwent the most dramatic changes during the adaptation (Table 2). To further explore the changing profile of gene expression level, up-regulated and down-regulated genes were analyzed separately. Interestingly, a large portion of genes involved in the myosin complex (11.5%) and motor activity (14.3%), which is related to muscle contraction, were up-regulated significantly (Fig. 2c). And the only two genes in troponin complex and one gene in skeletal muscle myosin thick filament were all significantly un-regulated (Table 2 and Supplementary Table S1). A large portion of genes in carbohydrate utilization were also up-regulated after the adaptation to tobacco plant (Fig. 2b). Myosin complex and motor activity could contribute to muscle contraction through promoting the sliding between two actin filaments^18^. And carbohydrate metabolism might be responsible for supplying energy for muscle contraction. Glandular trichomes of tobacco plant can secrete oils and polysaccharides, producing resin-like sticky gum on the leaves, which can provide mechanical or chemical protections against insect pests^19^. Our data suggested that stronger muscle might help whitefly overcome physical barriers on tobacco plant.

**Table 2:** GO terms enriched with differentially expressed genes.

GO Term	P-value	Count^1^	Size^2^	GO Description
GO:0008152	7.73E-05	16	484	metabolic process
GO:0005861	0.000107	2	2	troponin complex
GO:0090599	0.000126	3	9	alpha-glucosidase activity
GO:0016459	0.002818	3	26	myosin complex
GO:0043169	0.003003	3	25	cation binding
GO:0005506	0.003719	5	90	iron ion binding
GO:0016831	0.004064	2	9	carboxy-lyase activity
GO:0055114	0.005323	9	288	oxidation-reduction process
GO:0016787	0.007952	9	304	hydrolase activity
GO:0009058	0.008411	2	12	biosynthetic process
GO:0019752	0.008988	1	1	carboxylic acid metabolic process
GO:0003774	0.009466	2	14	motor activity

^1^The number of differentially expressed genes.

^2^The total number of genes in a GO term.

### Analyses of whitefly body volume and muscle content

**Figure 3:**
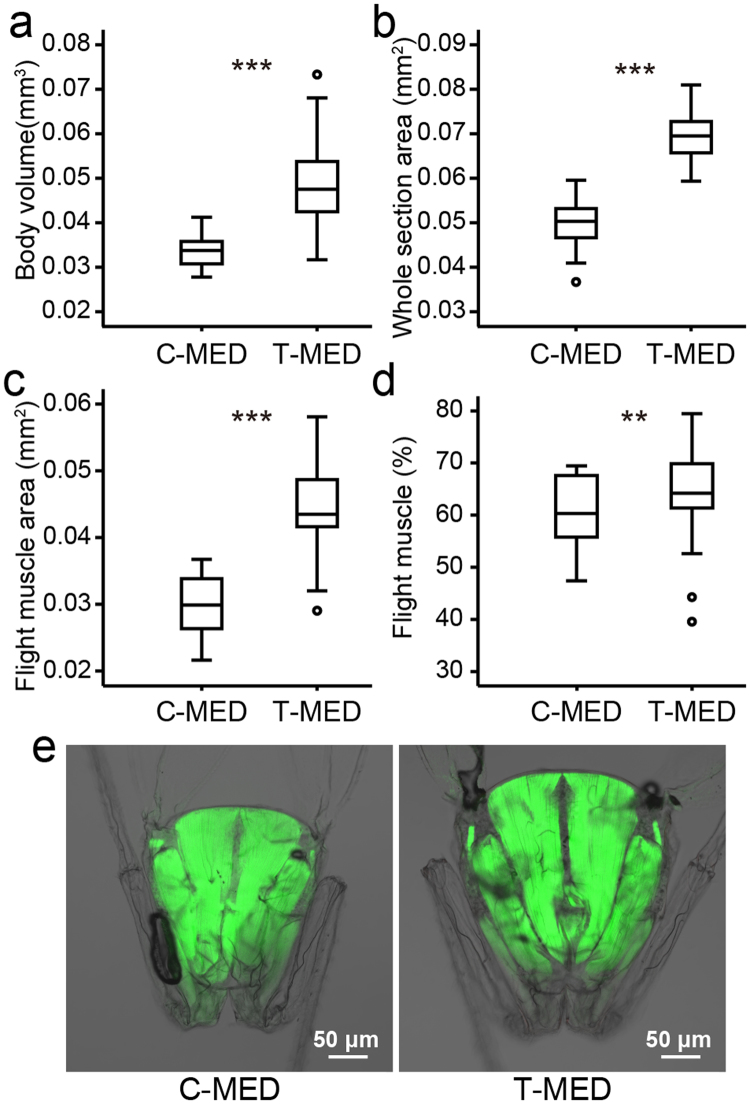
Comparing the body volume and flight muscle of the two whitefly populations. Whitefly reared on tobacco or cotton for 10 generations were used for this study. Box plots were used to show the body volume (**a**), area of the whole section of pterothorax (**b**), area of flight muscle (**c**) and the ratio of flight muscle of whole section (**d**) of newly emerged whitefly. Open circles indicate outliers. (**e**) Image showing the pterothorax of whitefly corresponding to the individual that exhibit the near mean flight muscle area (0.030 mm^2^ for cotton population; 0.429 mm^2^ for tobacco population). Flight muscle (green) were labeled with Alex 647 conjugated phalloidin. n = 30. Statistically significant difference: **P < 0.01, ***P < 0.001.

To further explore the biological meaning of the higher expression of genes involved in myosin complex and motor activity, we compared the body volume and muscle content of the two whitefly populations after 10 generations. The body volume of whitefly was estimated by the body length and width. The whitefly population on tobacco showed significantly larger body volume than the population on cotton (Fig. 3a; two-tailed independent-samples t-test, n = 30, P < 0.001). To compare the muscle content of the two whitefly populations, pterothorax was dissected and observed under confocal microscope. Flight muscle was labeled by fluorescein conjugated phalloidin. Areas of the whole pterothorax section and flight muscle were captured and measured. The area of the whole section (Fig. 3b) and the area of flight muscle (Fig. 3c) were significantly larger in T-MED than C-MED (two-tailed independent-samples t-test, n = 30, P < 0.001). The percentage of flight muscle area of the whole section area was significantly higher in T-MED than C-MED (Fig. 3d; two-tailed independent-samples t-test, n = 30, P < 0.01). Figure 3e shows the average flight muscle area of the two whitefly populations. The higher percentage of area occupied by myofibrils indicates stronger muscle^20^. This result suggested that T-MED might have stronger muscle and it might help whitefly overcome physical barriers on leaf surface.

### Effect of plant trichomes against whitefly

**Figure 4:**
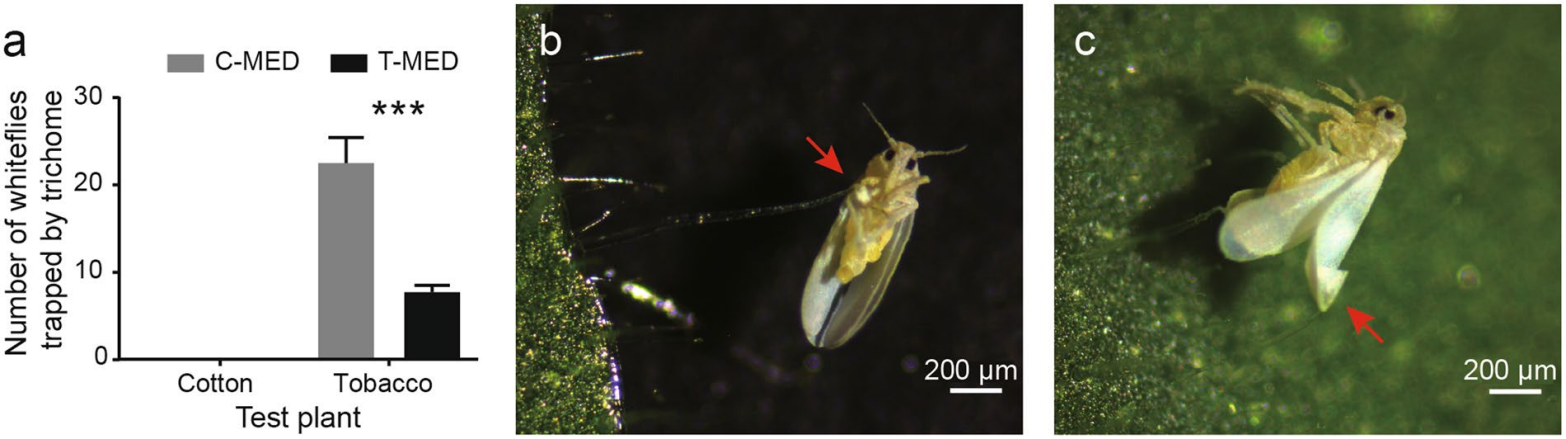
The influence of tobacco trichome on two whitefly populations. (**a**) Whitefly reared on tobacco or cotton for 10 generations were used to study their performances on tobacco plants. The number of whiteflies that was trapped by the trichomes on tobacco or cotton were confirmed and counted under stereoscopic microscope. Plane (**b**) and (**c**) show the whiteflies that were trapped by the trichomes on tobacco plant. Data shown are mean ± SE. n = 8. Statistically significant difference: ***P < 0.001.

**Figure 5:**
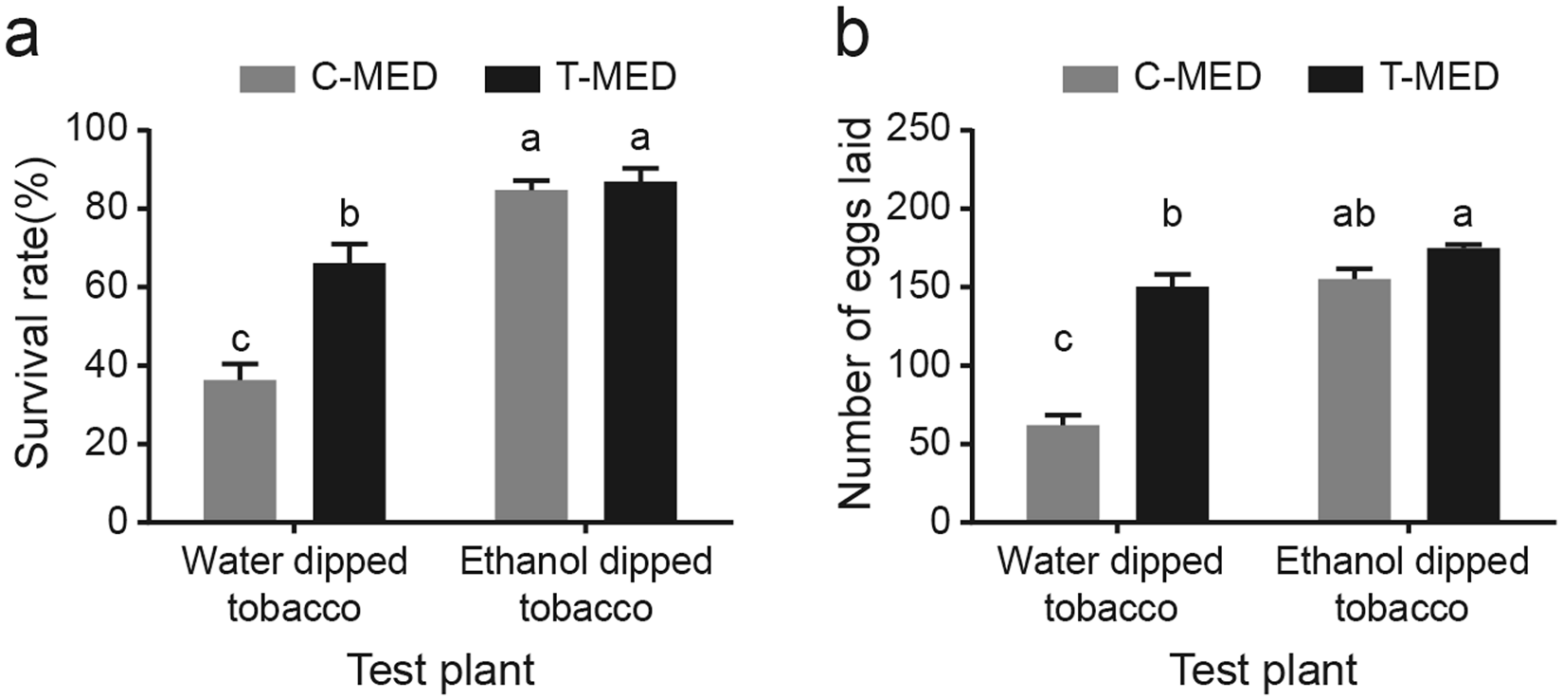
Effect of trichome exudate removal on whiteflies. The sticky exudate of tobacco trichomes were removed by ethanol and tobacco treated with water were included as control. Then the survival rate (**a**) and fecundity (**b**) of both populations were examined on them. Data shown are mean ± SE. n = 30. Different letters indicate significant differences between treatments at P < 0.05.

Next, we tested the effect of trichomes on whitefly after 10 generations. The numbers of whiteflies that were trapped by tobacco trichomes were significantly higher in C-MED than T-MED. However, whiteflies were never found to be trapped by the trichomes on cotton (Fig. 4a). In most conditions, the wings and legs of whiteflies were trapped by the head of glandular trichomes which could excrete or accumulate lipophilic substance, proteins, and secondary metabolites (Fig. 4b,c). Mostly, the ventral of whiteflies rather than the dorsal were stuck to the head of trichomes and some of the whiteflies trapped by trichomes were found to be alive and struggling. It indicated that whiteflies might first be trapped by trichomes and then die of starvation. The removal of trichome exudate from tobacco resulted in significant increase in the survival rate and fecundity of both T-MED and C-MED (Fig. 5). And both populations showed similar performances on tobacco plants after the removal (Fig. 5). Over all, these results suggested that trichomes of tobacco plant might be an important defense mechanism against whitefly, and whitefly could effectively circumvent it after adaptation for several generations probably through developing larger volume of body and muscle.

### Expression profile of endosymbionts

The differentially expressed genes in endosymbionts of whitefly were also studied by transcriptome analyses. The expression level of genes in *Portiera* (Supplementary Table S2) and *Hamiltonella* (Supplementary Table S3) was analyzed after normalized by the total reads mapped to their genomes, respectively. Gene with more than 2 fold change was not observed except a small heat shock protein, a lipoprotein and a hypothetical protein in *Hamiltonella*.

## Discussion

The fast and widespread invasions of invasive whiteflies have been referred to be related to their wide range of host plants^13–15^. Plants can produce toxins to disrupt physiological processes in the insect, which play an important role in restricting insect herbivores^3,21,22^. Previous study showed that MEAM1 and MED whitefly but not Asia II 3 could develop successfully on tobacco plant, and the higher activity of detoxification in invasive whiteflies might be responsible for this^17^. However, we found that after adaptation for 10 generations, the expression level of genes involved in detoxification, including cytochrome P450 monooxygenases, carboxylesterases, and glutathione S-transferases, did not change significantly, which is similar to the previous short-term study about host plant effect^16^.

RNA-Seq and image analyses showed that whiteflies had higher expression of genes involved in motor activity and increased muscle content after the adaptation to tobacco plant. It suggested that they might have strong muscle and be easier to overcome physical barriers on plant surface such as spines, setae and trichomes. Adapting to tobacco plant by strengthening their muscle is consistent with previous studies about the resistance of plant to whitefly. Large scale screen and morphological analysis of tomato plants indicated that the presence of glandular trichomes type VIc can enhance their resistance by entrapping whitefly^23,24^. Quantitative trait locus analysis for whitefly resistance in tomato found that there could be some linkage between the resistance and the density of type IV and type VI trichomes^25,26^. Electrical penetration graph study also revealed that whitefly spent more time in non-probing activities on tomato plant with the presence of type IV glandular trichomes^27^.

In contrast, several studies showed that higher oviposition rates occurred with dense trichomes on cotton, soybean and tomato^28–30^. It indicates that whitefly resistance may be associated with specific type of trichome. The head cells of tobacco trichomes are coated with sticky resin-like polysaccharides which can confer protection against insect pests^19,31^. We found that whiteflies of C-MED were easier to be trapped by the head of tobacco trichomes than T-MED and the removal of sticky exudate increased the survival rate and fecundity of both populations (Figs 4 and 5). Accordingly, sticky exudate was not found on cotton trichomes and whiteflies were never found to be trapped by them (Fig. 4).

These results indicated that tobacco trichomes and their sticky gum might hinder whiteflies from searching suitable feeding site and cause high mortality. And whiteflies might adapt to tobacco by increased muscle content and body mass. In addition, our results showed that the adaptation to tobacco did not influence whiteflies’ performance on cotton (Fig. 1). However, increased muscle content will consume more energy, and it may have adverse effects on whiteflies that have not been observed.

Endosymbionts play important roles in the life cycles of phloem-feeding insects^32,33^. Obligate symbiont *Portiera* of whitefly can produce essential amino acids and carotenoids for its host and facultative symbiont *Hamiltonella* may complete the synthesis of essential amino acid and provide vitamins^34,35^. They might help the adaptation of whitefly by providing nutrition that is rare in the phloem sap of tobacco plant. However, genes from both endosymbionts were expressed steadily after 10 generations on tobacco, which is consistent with the lack of cis-regulatory element in endosymbionts’ genome^36^. It suggested that endosymbionts were not involved in the adaptation process to tobacco plant. However, the expression profiles of endosymbiont genes under different conditions have not been well studied, and it cannot exclude the possibility that they change in other situation.

In summary, our study showed that MED whitefly could significantly improve its performance on tobacco after adaptation for several generations. Genes involved in myosin complex and motor activity were up-regulated significantly after rearing on tobacco for 10 generations. Glandular trichomes on tobacco plant might hinder whitefly from moving smoothly on the surface of tobacco plant and result in low survival rate, and whitefly might adapt to it via developing larger volume of insect body and muscle.

## Materials and Methods

### Whitefly rearing

The MED whiteflies were first collected on cayenne pepper from Zhejiang province of China, and maintained on cotton for 5–10 generations. Then, about 1,000 whiteflies was taken to establish two new separate cultures on cotton and tobacco respectively. Cotton *Gossypium hirsutum* (cv. Zhe-Mian 1793) and tobacco *Nicotiana tabacum* (cv. NC89) were used as the host plants for rearing MED whiteflies. All plants used in experiments grown to the four to six true leaf stage at the start. Whiteflies were maintained in climate chambers at 26 ± 1 °C, with a photoperiod of 14 h/10 h and 70 ± 10% relative humidity. Every three generations, the purity of the cultures was monitored by PCR-restriction fragment-length polymorphism analysis and sequencing of cytochrome oxidase I (COI, GenBank accession no. GQ371165) with the primer COI-F (5′-TTGATTTTTTGGTCATCCAGAAGT-3′) and primer COI-R (5′-TCCAATGCACTAATCTGCCATATTA-3′) as reported early^37^.

### Performance of whitefly on tobacco

Whitefly population maintained on cotton was transferred and reared on cotton or tobacco plants separately. After 1, 2, 5 and 10 generations, the two populations of whitefly were transferred onto new tobacco plants and their performances were examined on it. Four days prior to the bioassay, for each of the two whitefly populations, cotton or tobacco with red-eye pupae were placed into a new insect proof cage after removal of all the adults on the plants. Forty-eight hours later, the newly-emerged adults were used for experiments. Micro-cages of which the diameter is about 3.0 cm were used to keep whiteflies on the abaxial surface of leaves. For each test, 5 males and 5 females were collected randomly and transferred into a micro-cage. Thirty micro-cages (replicates) were done for each population. To minimize the probable effect of different leaves, two micro-cages were placed symmetrically on each leaf, one with adults from tobacco and other with adults from cotton. The adults were kept on the leaves for 7 days for them to oviposit. The live and dead adults and numbers of eggs laid in each micro-cage were then counted. To monitor the sex ratio of their progeny, similar process was done except that adults were removed after 7 days and micro-cages were left on leaves to let whiteflies emerge in them. Newly emerged adults in each micro-cage were collected and sexed after 30 days.

### Sample preparation and Illumina sequencing

After rearing on cotton or tobacco for 10 generations, 200 adult whiteflies emerged 1–3 days were collected for each sample, frozen in liquid nitrogen and stored at −80 °C till further use. Total RNA was isolated from each sample separately using SV Total RNA Isolation System (Promega, USA) following the manufacturer’s protocol. The integrity of the RNA samples was analysed on the 2100 Bioanalyzer with RNA Nano 6000 Assay Kit (Agilent Technologies, USA). Three biological replicates for both whitefly populations were conducted independently and then be pooled for RNA sequencing. To detect the potential sequence variants and transcripts from endosymbionts, deep sequencing and rRNA removal kit were recruited. The mitochondrial, endosymbiont and whitefly rRNAs were removed using Ribo-Zero Gold rRNA Removal Kit (Illumina, USA) following the manufacturer’s protocol. The mRNA was fragmented and cDNA library was prepared using Tru Seq RNA sample preparation kit v2 (Illumina, USA) following the manufacturer’s protocol. The library was sequenced, in a paired end 100 base run on the Illumina HiSeq. 2000 platform following the standard protocols provided by the manufacturer.

### Gene expression analysis

Transcriptome sequencing gave 217 M reads for C-MED and 224 M reads for T-MED in a single lane, in which more than 86% bases have 30 or greater PHRED quality score. After the filtering of reads containing adapter and poly-N and low quality reads, 186 M (85.7%) and 163 M (72.7%) reads were left, which is within the acceptable range recommended (Table 1). Clean sequencing data is available at the NCBI Short Read Archive (SRA) database (accession number: SRR4039449, SRR4039450). To calculate the gene expression level of whitefly, reads were mapped to the reference genome of whitefly MEAM1 (accession number: NC_006279.1) by Tophat2 with the parameters as follows:–read-mismatches 3–read-gap-length 3–read-edit-dist 3–no-mixed–no-discordant -p 6^38^. A maximum of 3 mismatches was allowed due to the sequence difference between whitefly MED and whitefly MEAM1. Further, the abundance of transcripts were estimated by Cufflinks-Cuffquant with default parameter and the existing gene annotations were used. Tests for differential expression between two whitefly populations were performed by Cufflinks-Cuffdiff with default parameters^39^. The number of read count was adjusted with the length of exon and the number of mapped fragments to calculate fragment per kilobase per million mapped reads (FPKM). The reference genome and annotations were downloaded from ftp//whiteflygenomics.org/.

To calculate the gene expression level of endosymbionts, reads were mapped to the genome of *Portiera* and *Hamiltonella* respectively by bowtie2 with the parameters as follows:–very-sensitive–phred33 -N 1. Then the mapped read of each gene was counted by HTseq with default parameters^40^. Differentially expressed genes were analyzed by DEGseq with default parameter^41^.

### Real-time quantitative PCR analysis

Quantitative real time PCR was recruited to further validate the expression of selected genes and density of endosymbionts. Whitefly reared on tobacco or cotton for 10 generations were collected and frozen in liquid nitrogen. There were three biological replications for each population and 200 whiteflies for each replication. Total RNA were isolated as described before. Reverse transcription was conducted with PrimeScript RT reagent Kit (TaKaRa, Japan) following the manufacturer’s protocol. qPCR were performed on CFX connect Real-Time PCR system (Bio-Rad, USA) using the SYBR Premix Ex Taq TM II (TaKaRa, Japan) following the standard protocol described. Ribosomal protein L32 was chosen as endogenous reference gene due to its stable expression across different physiological and developmental stages^42^. Ribosomal protein L32 was also shown to be stably expressed in transcriptome analyses between two populations. The relative abundance of transcripts was calculated basing on 2−ΔΔCt method. The reaction mixture (20 μl) consisted of cDNA (2 μl), specific primers (8 pmole) and 2X SYBR Green PCR Master Mix (10 μl). For each gene, three biological replicates and three technological replicates were done. Primers used in this study were listed in Supplementary Table S4.

### Identification of statistically enriched pathways

GO enrichment analysis of the differentially expressed genes was conducted basing on Wallenius non-central hyper-geometric distribution, which can adjust for gene length bias^43^. P-value < 0.01 was set as the threshold for enrichment.

### Image analysis of flight muscle

Only whiteflies within the 2-day period after emergence were used in this study to exclude the effects of experience on host plants on flight muscle development. After adaptation for 10 generations, whiteflies that reared on tobacco and cotton were collected and fixed in 4% paraformaldehyde for 2 hours after a quick wash in ethanol. The body volume was calculated by the formula: *V* = *πab* ^2^/6, where *a* is body length and *b* is width. Then the last pterothorax was dissected and permeabilized with 0.5% Triton X-100 (Thermo Fisher, USA) for 30 min. The tissues were subsequently incubated with Alex 647 conjugated phalloidin (Thermo Fisher, USA) for 2 hours and mounted in glycerin for view. Pterothorax of whiteflies were captured with the Zeiss LSM780 confocal microscope (Zeiss, Germany) and the optical sections with the maximum flight muscle area were chosen for each whitefly. The area of flight muscle, the area of whole section and the percentage of flight muscle area in the whole section area were measured with IMAGEJ.

### Effect of trichomes on protection against whiteflies

To monitor the effect of trichomes on protection against whiteflies, whitefly reared on tobacco for 10 generations and whitefly reared on cotton were used to study their performances on tobacco and cotton. For each test, 50 newly-emerged whiteflies were released in an insect proof cage with one tobacco or cotton plant. Seven days later, the numbers of whiteflies that were trapped by trichomes were counted under stereomicroscope. Eight replicates were done independently for each population.

To remove the sticky exudate of trichomes, the leaves of tobacco were first dipped briefly in 95% ethanol and then washed with water. The tobacco leaves dipped briefly in water were included as control. Evaporation of the ethanol and water was hastened with the use of a blow drier.

### Statistical analysis

The survival rates, sex ratio, and percentage of flight muscle area in the whole section area were arcsine square root transformed for statistical analysis and back-transformed for presentation in the figures. Two-tailed Paired-samples t-test was applied to compare the survival rate, sex ratio, and number of eggs laid between different populations of whitefly. Comparison of flight muscle area and whole section area were performed by two-tailed independent-samples t-test. Comparison of number of whiteflies trapped by trichomes were conducted with Mann-Whitney U test. Survival rate, sex ratio, number of eggs laid, flight muscle area and whole section area were showed to be normally distributed using Kolmogorov-Smirnov and Shapiro-Wilk test. P-value < 0.05 was considered as the threshold for significant difference. All the statistical analyses were performed with SPSS 20.0 (SPSS Inc., USA).

## Electronic supplementary material

Figure S1

Table S1

Table S2

Table S3

Table S4
