# Supplementary material for: Transcriptome analyses suggest a novel hypothesis for whitefly adaptation to tobacco
Source: Sci Rep. 2017 Sep 21;7:12102. doi: 10.1038/s41598-017-12387-3 (PMC5608870; doi:10.1038/s41598-017-12387-3)
Supplement: Supplementary file 1 — Figure S1 [file 41598_2017_12387_MOESM1_ESM.doc]

**Supplementary Information for:**

**Transcriptome analyses suggest a novel hypothesis for whitefly adaptation to tobacco**

Wen-Qiang Xia, Xin-Ru Wang, Yan Liang, Shu-Sheng Liu and Xiao-Wei Wang*

Ministry of Agriculture Key Laboratory of Agricultural Entomology, Institute of Insect Sciences, Zhejiang University, Hangzhou 310058, China

Correspondence should be addressed to Xiao-Wei Wang. Address: Ministry of Agriculture Key Laboratory of Agricultural Entomology, Institute of Insect Sciences, Zhejiang University, Hangzhou 310058, China. Telephone: 86-571-88982435. E-mail: [xwwang@zju.edu.cn](mailto:xwwang@zju.edu.cn)

**Supplementary Figure S1. qPCR validation of gene expression.** 10 up-regulated, 10 down-regulated and 10 non-differentially expressed genes were selected randomly and analyzed by quantitative real-time PCR following reverse transcript.

**Table S1.** Expression level of genes on the genome of whitefly.

**Table S2.** Expression level of genes on the genome of *Portiera*.

**Table S3.** Expression level of genes on the genome of *Hamiltonella*.

**Table S4.** Information for the primers used in qPCR validation.

**Figure S1.**


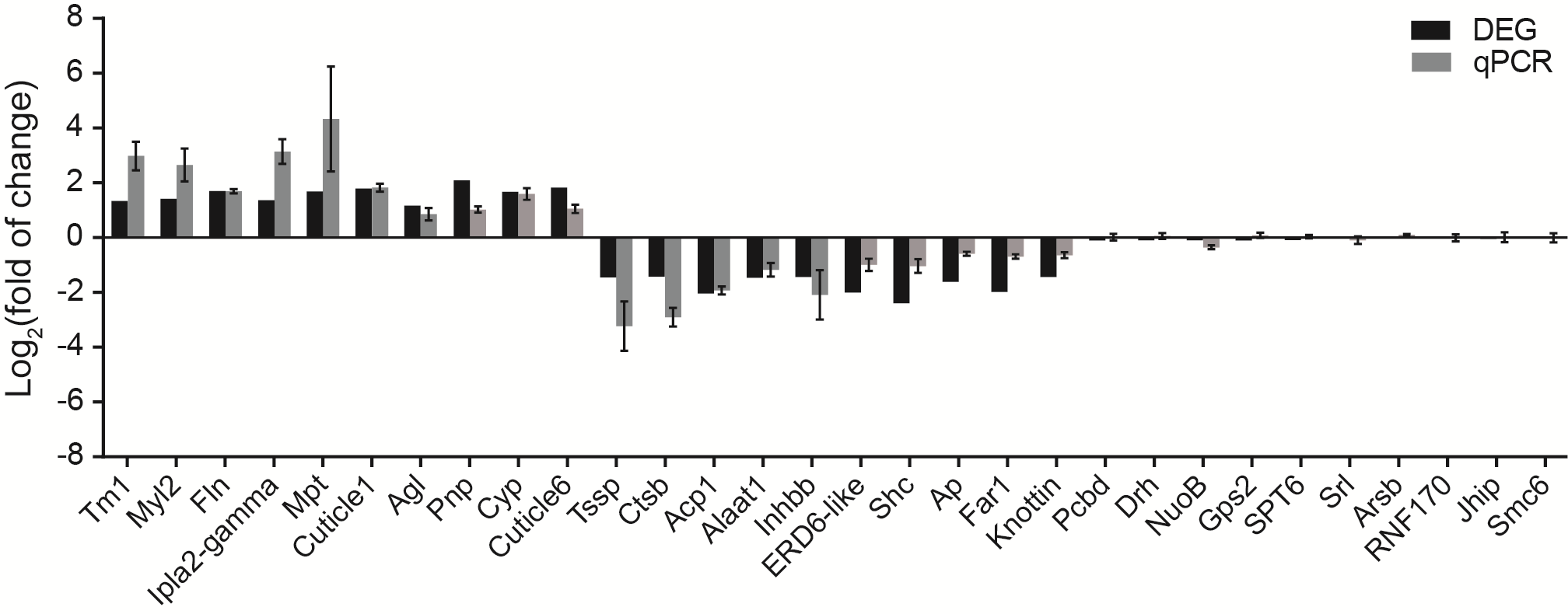


**Fig. S1. qPCR validation of gene expression.** Populations of whiteflies reared on cotton and tobacco for ten generations were employed. Totally 30 genes including 10 up-regulated, 10 down-regulated and 10 non-differentially expressed genes were selected randomly and analyzed by quantitative real-time PCR following reverse transcript. Three biological replicates were done for each gene. Data shown are mean ± SE. Fold change of these genes in qPCR analysis were mostly consistent with transcriptome analyses. Abbreviation for gene names: tropomyosin 2 (Tm2), myosin 2 light chain (Myl2), flightin (Fln), calcium-independent phospholipase A2-gamma (Ipla2-gamma), mitochondrial phosphate carrier protein (Mpt), cuticle protein 1 (Cuticle1), alpha-glucosidase (Agl), purine nucleoside phosphorylase (Pnp), cytochrome P450 (Cyp), cuticle protein 6 (Cuticle6), thymus-specific serine protease (Tssp), cathepsin B (Ctsb), acid phosphatase-1 (Acp1), alanine aminotransferase 1 (Alaat1), inhibin beta B chain (Inhbb), sugar transporter ERD6-like 6 (ERD6-like), squalene-hopene cyclase (Shc), aromatic peroxygenase (Ap), fatty acyl-CoA reductase 1 (Far1), pterin-4-alpha-carbinolamine dehydratase (Pcbd), ATP-dependent RNA helicase (Drh), NADH-quinone oxidoreductase subunit B (NuoB), G protein pathway suppressor 2 (Gps2), transcription elongation factor SPT6 (SPT6), sarcalumenin (Srl), arylsulfatase B (Arsb), E3 ubiquitin-protein ligase RNF170 (RNF170), juvenile hormone-inducible protein (Jhip), structural maintenance of chromosomes protein 6 (Smc6).
